# Multisensorial Assessment of Laser Effects on Shellac Applied on Wall Paintings

**DOI:** 10.3390/s21103354

**Published:** 2021-05-12

**Authors:** Jana Striova, Raffaella Fontana, Ilaria Barbetti, Luca Pezzati, Annamaria Fedele, Cristiano Riminesi

**Affiliations:** 1 National Research Council, INO, Largo E. Fermi 6, 50125 Firenze, Italy; jana.striova@cnr.it (J.S.); raffaella.fontana@cnr.it (R.F.); afedele32@gmail.com (A.F.); 2 Soprintendenza Archeologia, Belle Arti e Paesaggio per le Province di Pisa e Livorno, Lungarno A. Pacinotti 46, 56126 Pisa, Italy; ilaria.barbetti@beniculturali.it; 3 National Research Council, ISPC, Via Madonna del Piano 10, 50019 Firenze, Italy; cristiano.riminesi@cnr.it

**Keywords:** laser cleaning, shellac coating, wall painting, thermal infrared imaging, Ho:YAG laser

## Abstract

The assessment of five different laser treatments in the conservation of wall paintings was devised on the basis of the surface temperature monitoring by infrared thermography (IRT), ultraviolet-induced fluorescence-visible (UV-VIS) imaging, and optical coherence tomography (OCT). A series of yttrium-aluminum-garnet (YAG) lasers were tested for removal of shellac layers from wall painting mock-ups. The mock-ups were realized as *buon fresco* with different mineral based pigments (earths and iron oxide) on a lime- and sand-based mortar. After the carbonatation process, all the samples were treated with shellac (5% in ethanol). The effects of neodymium (Nd):YAG, holmium (Ho):YAG, and erbium (Er):YAG laser sources, in different operative modes, on average temperature of the surface, color, and morphology were inspected with complementary sensors. The results show the necessity to adopt a combined approach in establishing safe laser operating conditions to avoid any undesired effects induced on the artefacts by the laser treatments. We demonstrate, for the first time, the performance of the Ho:YAG laser in the removal of a conservation treatment.

## 1. Introduction

The cleaning is an irreversible restoration intervention on cultural heritage assets involving the removal of unwanted substances generally deposited on (or embedded into) the surface of the object. The undesirable materials may include alteration products of the constituent materials, dust, salts efflorescence, soluble and insoluble concretions due to the alteration of organic materials or crystallization of inorganic materials, layers of products applied in the past, altered and no longer functioning [1]. The principal task of cleaning is the removal of harmful or potentially dangerous substances. The decision as to what material should be preserved or removed is crucial for planning the restoration, along with the definition of the most appropriate methods to carry out a selective, effective and safe cleaning. These choices imply a proper knowledge of the artwork’s materials and history, as well as of the expected performance of the cleaning methods. A cultural heritage object is a unique and irreplaceable testimony of cultural and social identity; thus, the aim of cleaning must be first of all its preservation. The target for the restorer is to exploit the most suitable techniques and their combination in order to guarantee the best possible results in terms of cleaning intervention.

Mechanical, chemical, and laser methods can be involved in the cleaning of wall paintings, either standalone or in combination. The mechanical cleaning entails tools, such as brushes, soft tires, vacuum cleaners, scalpels, and abrasive materials, for the removal, thinning or abrasion of the undesired material [2]. The chemical cleaning exploits solvents or reagents capable of modifying the state of aggregation of the substances to be removed. A variety of them are used in the form of pure solvent, such as: water or organic solvents; aqueous solutions; acids and bases with controlled pH; complexing and chelating agents; surfactants; multicomponent formulations containing ion-exchange resins; emulsions; artificial saliva, or solvent surfactants gels [3]. The products can be applied directly, by cotton swabs, a sheet of paper, cellulose-based poultice, or by gels. The latter approach allows the controlling of solvent release and of its penetration depth in the substrate. Then, the removal of the solubilized or swelled material can be performed by means of physical action. The laser techniques have gradually gained a prominent position thanks to scientific studies proving their potential for a selective, gradual, and accurate removal [4]. The interaction mechanism (photoacoustic or thermal) depends on both the material characteristics and the lasing parameters. The correct selection of these latter, that should keep the laser power below the damage threshold, is indeed crucial for a safe and efficient treatment [5,6,7,8].

A rigorous assessment of the cleaning outcomes is crucial to avoid damaging the substrate, and in particular the paint layers. Scientific investigations have been performed before, during, or after the cleaning intervention by measuring chemical and physical properties, such as molecular composition, color, surface roughness, and by quantifying the thickness of the preserved patina using various non-invasive diagnostic tools [9,10,11,12,13,14]. The heating induced by laser treatments is another important effect to be kept under control, especially when treating materials with a high optical absorption coefficient in relation to the laser wavelength, as it often occurs during interventions on thermosensitive pigments and materials [10,15].

Laser treatment is nowadays largely diffused for cleaning different materials: metals, paintings, and wall paintings [8,16,17,18]. A preliminary setting of the laser parameters and a real-time control of the temperature of the surface exposed to laser radiation should prevent both instant and long-term damages [4,19]. Indeed, the interaction mechanisms between the laser radiation and the materials constituting the artworks are complex due to the stratified systems composed by diverse intercorrelated organic and inorganic materials, all potentially affected by the laser radiation during the treatment. A preliminary stage of diagnostics is mandatory to define the safe treatment protocol and to prevent undesired effects and damages. Conservation interventions may be undertaken with various laser sources, the Nd:YAG and Er:YAG lasers being the most diffused ones. The desired outcome can be tuned by a proper setting of parameters, such as the wavelength, the fluency, the pulse duration, the repetition rate (frequency), and the time of exposure [6,19]. However, laser absorption by the material constituting the wall painting contributes to raising the temperatures of both the surface and the underlying volume. Previous studies proposed the detection of the temperature increase upon the Er:YAG laser irradiation in medical and commercial field by exploiting thermocouples [20,21]. A similar approach was applied to monitor the effects of the Er:YAG laser in the cultural heritage field by De Cruz et al. [22]. Temperature monitoring following laser irradiation in Raman spectroscopy has been devised by Osticioli et al. [23]. The authors used a thermopile sensor (response time of 1.3 s, absolute accuracy of 0.5 °C at 25 °C, 10° field of view) and an optical circuit to monitor the effects of the laser radiation focused on the target surface. 

Here, we describe a multisensorial approach to monitor the effects on the laser-treated surface combining infrared thermal imaging (IRT), optical coherence tomography (OCT), and UV-VIS imaging. We tested the efficiency of high-speed IRT in revealing the temperature of the surface under treatment. Several laser sources, in particular Nd:YAG, Er:YAG, and Ho:YAG, were exploited in removal of shellac layer from the wall painting mock-ups. Their performances in terms of induced effects were compared in relation to the operational mode of lasers (wavelength, energy, fluency, pulse duration, and frequency). While the Nd:YAG and Er:YAG lasers are routinely applied in conservation interventions [5,6,7,8,12,13,17,18], the Ho:YAG laser emitting at 2100 nm is used up-to-now mainly in medical treatments, specifically in intracorporeal lithotripsy [24,25,26]. To the best of our knowledge, this is the first time that the use of a Ho:YAG laser to remove conservation treatments is reported. Depending on the operative conditions, its potential advantage could dwell in the sub-ablative action that mechanically compromises the material to be subsequently removed by a cotton swab. The free-running pulse emitted by the Ho:YAG laser acts mainly through the photothermal mechanism leading to the material expansion or vaporization in a way similar to that of the Er:YAG laser. Indeed, the radiation at the fundamental wavelength (2.1 μm) is weakly coupled with the -OH or -NH combination modes as compared with the Er:YAG (2.9 μm) radiation which is in resonance with these stretching modes. These wavelengths are also attractive due to the eye-safe nature of the radiation. This could be seen as a major advantage over the excimer lasers emitting, with different pulse duration, in the UV region and that prove extremely efficient in thinning the varnish layers by exploiting their high absorption at those wavelengths [27,28,29,30]. Finally, the shellac and other organic compounds exhibit poor absorption at 1064 nm thus compromising the cleaning efficiency of the Nd:YAG laser at its fundamental emission.

## 2. Materials and Methods

In this section the samples used for the experiments are described along with the laser-removal approaches and the diagnostic techniques employed to assess the surface chenges.

### 2.1. Samples

**Figure 1:**
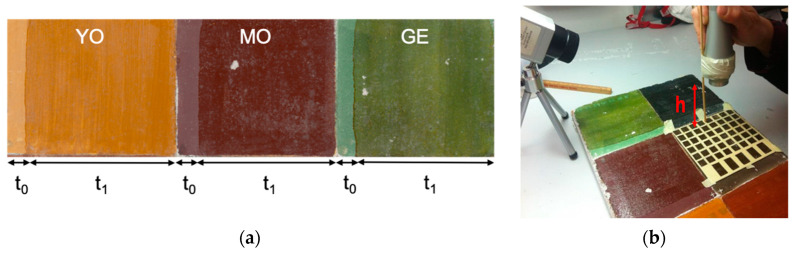
(**a**) *Affresco* mock-up (45 × 12 cm^2^) subdivided in 3 areas (15 × 12 cm^2^). From left to right: yellow ochre (YO), *morellone* (MO), green earth (GE) pigments; t_0_—reference area, t_1_—treatment with shellac; (**b**) set-up scheme for temperature monitoring: IR camera is positioned at 45° and laser at 90° with respect to the sample surface.

The mock samples for the tests simulating the *buon fresco* technique were prepared using calcium hydroxide mixed with sand at a ratio of 1:3 for the first mortar layer (*arriccio*) and 1:2 for the final mortar layer (*intonachino*). The fresh mortar surface was then polished with a trowel and painted with yellow ochres, *morellone* (a mixture of red and black pigments), and green earth (Figure 1, Table 1). For simulating the pictorial layer, we chose the most common inorganic and organic pigments in the medieval and renaissance palette for wall paintings. The pigments are based on iron oxides or hydroxide, complex aluminosilicate minerals, and amorphous carbon.

An artificial patina was then applied in the zone referred to as t_1_, composed of 5 (*w/v*) % shellac in ethyl alcohol, a natural fixative widely used in the past that over time becomes progressively less soluble and dark. In each of the pigmented zones, a reference area (t_0_) was left uncovered by the patina to facilitate analytical checks (Figure 1a).

### 2.2. Laser Treatments

Preliminary tests were performed for each type of pigment by setting the optimal laser parameters (energy—*E*; fluence—*F*; frequency—*f*; pulse width—τ) for removing or thinning the varnish layer. For each pigment, 18 areas were selected for the preliminary tests (1 × 1 cm^2^) to define the conditions for five tests (1 × 2 cm^2^) with the five different laser types, as described in Table 2 (I–V). The abbreviations QS, LQS, SFR stand, respectively, for Q-switched, long Q-switched, short-free running pulse durations. The fluence was calculated according to

*F* = *E/A*, (1)

where *A* is the area of laser beam.

A mask, made of PVC and masking tape, was used to localize the treatment areas in the same position on each pigment to allow a fast and simply performance comparison (Figure 1b). On the mask, each hole corresponding to a treated area was then unambiguously labeled. The selected area was preventively wet with demineralized water, then it was laser irradiated for five times, and successively a mild mechanical action was applied by rolling on the surface for five times a cotton swab moistened in ethyl alcohol. 

### 2.3. IR Thermography to Assess the Surface Temperature

During the cleaning, the temperature on the treated area was monitored in real-time by using an infrared (IR) camera, the Optris PI 450, a high resolution and multi-purpose infrared thermal imaging camera. The camera is equipped with a 9–14 μm band-pass filter and it has an average sensitivity of 0.03 °C in all its temperature-measuring range (from −20 °C to 900 °C). The camera framerate is 80 fps, corresponding to a sampling time τ = 12.5 msec. The recording of the temperature during the laser treatment was performed using a common photographic mini-tripod for positioning the camera (Figure 1b) at 45° and 25 cm of distance. The average temperature values refer to the 1 × 2 cm^2^ area under the cleaning for a time interval of 60 s. It may be useful to note that the sampling time of the IR camera (12.5 ms) was not synchronized with the laser pulse duration (from 15 ns to 500 μs) and the laser repetition rate (5 or 10 Hz). The acquired thermal images were recorded on a PC and processed in real-time through a dedicated software. The statistical information about the temperature was extracted in real-time for the selected area and plotted during the treatment.

### 2.4. UV-VIS-NIR Imaging and Color Measurements

**Figure 2:**
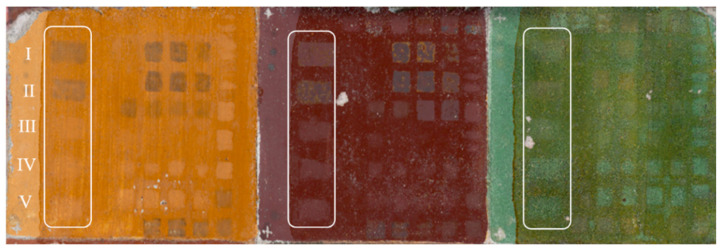
RGB image of the mock-up: white rectangles denote 1 × 2 cm^2^ zones of I–V laser tests as reported in Table 2, and are referred to as time 2 (t_2_). Other zones report the preliminary laser tests.

The specimen was examined by the visible-near-infrared (VIS-NIR) multispectral scanner described in detail elsewhere [31]. The scanner operates in the visible and the near-infrared regions (from 400 nm to 2500 nm), with a spectral resolution of 20–30 nm and 50–100 nm, respectively. Acquisitions were performed at time 0 (t_0_)— painted surface, at time 1 (t_1_)—after shellac application (Figure 1a) and at time 2 (t_2_)—after laser treatment (Figure 2). The RGB and the CIELab 1931 images (CIELAB 1931, Commission Internationale de l’Eclairage) were both calculated from the reflectance values of the VIS channels, using the standard D65 illuminant and the observer at 2°. The L*a*b* coordinates were used to calculate the color change ΔE, induced by both the shellac application (t_1_−t_0_) and the laser irradiation (t_2_−t_0_), according to the equation [32].

(2) $$\Delta E\;=\sqrt{\Delta L^{*2}+\Delta a^{*2}+\Delta b^{*2}},\;$$

where

(3) $$\Delta L^{*}=L_{x}^{*}-{\;L}_{t0}^{*},\;\Delta a^{*}=a_{x}^{*}-a_{t0}^{*},\;\Delta b^{*}=b_{x}^{*}-b_{t0}^{*};$$

x = t_1_ refers to the color parameters of the surface treated with the shellac, while x = t_2_ refers to the surface treated with the laser irradiation. ΔL* value describes a change in brightness where the L* value ranges between 100 (white) and 0 (black). The a* and b* values represent the color directions: +a* is red, −a* green; +b* yellow and −b* blue, ranging between +60 and −60. The positive (negative) values of Δa* and Δb* indicate that the treated areas are, respectively, more red (green) and yellow (blue) than the reference surface. 

UV induced fluorescence images were acquired with a Nikon D750 camera, equipped with a 105 mm f/2.8 AF micro lens. A Wood’s lamp, placed at nearly 45° with respect to the surface, was used to excite the sample, with the camera set parallel to its surface. Due to the low visible emission, fluorescence measurements were performed under dark conditions.

### 2.5. OCT for Thickness and Morphologic Evaluation

A device for measuring spectral-domain OCT (Telesto II, Thorlabs) was used to monitor the thickness of the shellac varnish and the effects of the laser treatments. The instrument operates at 1300 nm (center wavelength) with axial and lateral resolution of 5.5 (in air) and 13.0 microns, respectively. OCT cross sections were acquired at the cleaned–uncleaned interface for all the tests and the pigmented layers. The data were elaborated with ImageJ software. The thickness of organic layer as measured by OCT (d_OCT_) was corrected for the refractive index *n* to achieve the real thickness estimate. Being *n* = 1.516 the refractive index of shellac [33,34], the real shellac thickness (d_r_) is given by d_r_ = d_OCT_/*n*. In total, 10 measurements for each mean value were considered and the standard deviation provides indication on the homogeneity of the treatment. The maximum and minimum thickness values (d_max_ and d_min_) were extracted from the tomograms as well.

## 3. Results

A series of preliminary treatment tests were performed to assess each laser’s best configuration for removing or thinning the shellac layer. These led to the definition of the best possible experimental lasing conditions which are reported in Table 2 and labelled I–V. Figure 2 shows the true-color image of the mock-up, following all the laser tests. The I–V tests are evidenced by the white rectangles. The image was produced by processing the visible region spectral dataset acquired with the VIS-NIR multispectral scanner, collected in a 45°/0° illumination/detection geometry, by using the standard D65 illuminant and the CIE 1931 standard observer.

### 3.1. Real-Time Temperature Monitoring

**Figure 3:**
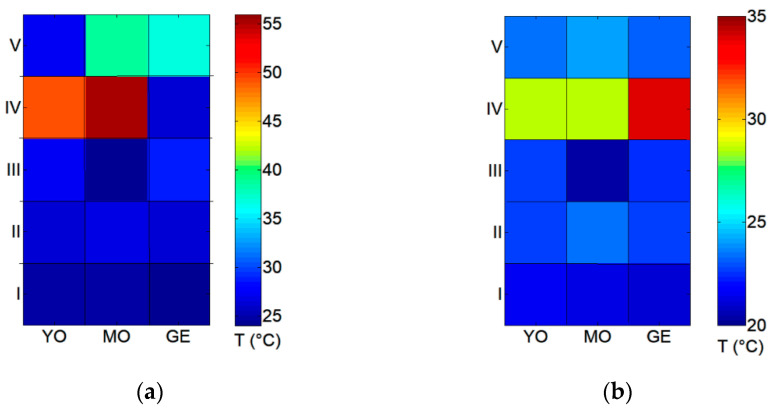
Plot of average temperatures measured with the infrared thermal camera as a function of laser treatment and pictorial layer of the irradiated 1 × 2 cm^2^ zones (**a**) in dry and (**b**) wet conditions.

The 1 × 2 cm^2^ sample surfaces were monitored in real-time with the infrared thermal camera for time intervals of 60 s, the time necessary to treat the whole test area. The graphs in Figure 3 show the average temperature values for the dry and wet irradiation conditions. The comparison demonstrates that the increase in surface temperature can be reduced by wetting the surface prior to the laser treatment. In wet conditions, used and discussed later in the text, the plot in Figure 3b shows that for all the laser treatments and the pictorial layers the average temperatures measured by the thermal infrared camera keep within the 20–25 °C interval. The exception is the Ho:YAG laser: higher average temperatures, in the 25–35 °C range, were actually registered during this treatment. Table 2 shows that the Ho:YAG laser uses the highest fluence (F = 10.2 J/cm^2^, more than 3× higher as compared to other lasers) to efficiently remove the shellac layer. For this laser, the radiation–material interaction is principally based on a thermal mechanism and the associated thermal effects generally increase as a function of fluence and diminish with the pulse duration [35].

While the monitoring of the temperature by IRT may reflect well the average thermal state of the sample under the treatment, it underestimates the maximum and instantaneous temperature value induced by laser radiation because the pulse durations are several orders of magnitude shorter than the camera response time (15 ns to 500 μs versus the 12.5 ms of the IRT sampling rate).

### 3.2. UV-VIS Imaging and Colorimetric Characterization

The RGB and the UV-induced fluorescence images, acquired after the cleaning tests, are shown in the following subsections for each paint layer. The colorimetric parameters referred to the reference surface (t_0_), to the surface with shellac treatment (t_1_), and to the laser-treated surface (t_2_) are also reported. The interpretation of the data considers both the observation of the visible and UV-induced fluorescence images, as well as the colorimetric coordinates and their changes as a function of the laser treatment. The organic layer exhibits UV-induced orangish fluorescence, which can be used to monitor the presence of shellac on the surface [36]. The data provided a reliable base for the evaluation of the laser effects and for further corroboration of the results by complementary optical measurements. 

#### 3.2.1. Yellow Ochre

**Figure 4:**
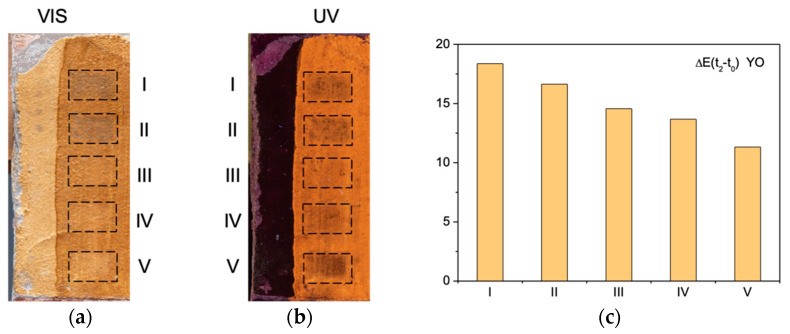
Yellow Ochre specimen as a function of laser treatment (I–V): (**a**) visible image; (**b**) UV-induced visible fluorescence; (**c**) bar plot of ΔE(t2–t0) values. Each tested area I–V refers to 1 × 2 cm^2^ area.

Figure 4a,b report images of the YO specimen after I–V laser treatments, respectively, in visible light and of UV-induced fluorescence. 

Table 3 documents the colorimetric coordinates of the painted surface at t_0_, t_1_, t_2_. The data show that the shellac application (t_1_) induced the diminishment of L* (ΔL_t1–t0_ = −15.0) and a significant increase in both a* and b* parameters (Δa_t1–t0_* = 8.0 and Δb_t1–t0_* = 12.0). 

Following the treatment by the Nd:YAG QS and LQS lasers emitting at their fundamental wavelength (1064 nm), the areas labeled I and II in Figure 4a exhibit darkening, evidenced by a decrease in L* values from 69.0 at t_0_ and 54.0 at t_1_ to, respectively, 51.0 and 53.0 at t_2_. As compared to t_0_, the values of b* show a decrease in the yellow component (Δb_t2−t0_* < 0) for both the Nd:YAG lasers (I and II), suggesting the modification of the pigment’s chromophore (yellow goethite and lepidocrocite, α-FeOOH and γ-FeOOH, respectively). As described in literature, these thermolabile chromophores may transform to dark maghemite (γ-Fe_2_O_3_), especially in presence of organic matter [33]. In either case, the presence of the dark material can be most likely attributed to the transformation of both the pigment and the residues of the organic layer. The colorimetric measurements evidenced that that the irradiation with a short-pulse Nd:YAG laser (I) induced greater extent of darkening than the long-pulse Nd:YAG (II) laser (ΔE(I) > ΔE(II)). Generally, photothermal effects diminish with longer laser pulse duration [35]. The opposite effect (an increase in L* values at t_2_ as compared to t_1_) is observed for the III, IV, and V areas suggesting a partial removal of shellac. The data suggest that the cleaning with the Er:YAG laser (V) has rendered the surface, by removing partially the shellac film, the most similar to its original colorimetric aspect at t_0_ (the smallest ΔL). As compared to t_0_, the values of b* show a decrease in the yellow component (Δb* < 0) for the Nd:YAG lasers (I and II) whereas Δb* > 0 for the other systems indicates the restoring of the original aspect of the paint layer, in the greatest extent for the V laser (b* at t_0_ = 39.3; t_2_ = 43.7). The UV-induced orangish fluorescence (Figure 2), typical for shellac, provides information on the presence, the distribution and the integrity of this organic film on the surface. The area V appears the darkest—the most similar to the reference paint layer—corroborating the hypothesis about the film removal. 

Figure 4c shows a plot of global color change (ΔE) values, calculated according to Equations (2) and (3), where the delta is referred to the surface after the laser treatment (t_2_)—reference surface (t_0_). The smaller the ΔE, the more similar the surface colorimetric properties. The values exhibit a clear trend (I > II > III > IV > V) suggesting that the cleaning with Er:YAG Long (V) laser is the most effective, followed by Ho:YAG Ablation (IV) and Nd:YAG SFR (III). On the other hand, the lasers Nd:YAG QS and LQS induced negative colorimetric changes that translated also into the high ΔE values, suggesting a damage of the substrate.

#### 3.2.2. Morellone

**Figure 5:**
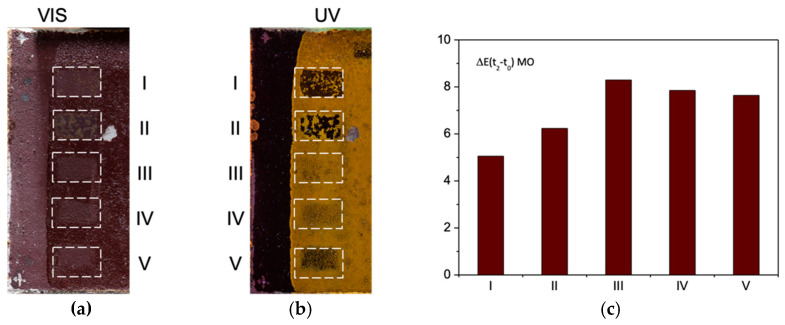
*Morellone* specimen as a function of laser treatment (I–V): (**a**) visible image; (**b**) UV-induced visible fluorescence; (**c**) bar plot of ΔE values. Each tested area I–V refers to 1 × 2 cm^2^ area.

Figure 5a,b report MO specimen images after laser treatment, respectively, in visible light and of UV-induced fluorescence. 

Table 4 documents the colorimetric coordinates at t_0_, t_1_, t_2_. The shellac application (t_1_) induced a decrease in L*, ΔL_t1−t0_ = −14.0, and an increase in a* and b* values (Δa_t1−t0_ = 7.0 and Δb_t1−t0_ = 11.7) which is comparable with the effect of shellac on YO.

The I–III laser treatments with Nd:YAG lasers were performed at the fundamental wavelength (1064 nm) by varying the pulse duration (from the shortest to the longest QS < LQS < SFR pulse width). Regarding the impact of laser treatment, the colorimetric values at t_2_ provide evidence of a very efficient cleaning by the Nd:YAG QS laser (I). Indeed, low differential values (t_2_–t_0_) (ΔL = −3.3, Δa = −1.0 and Δb = 3.7) suggest a very effective ablation of the shellac film. This was achieved, probably, through the photomechanical action of the very short-pulsed laser (QS, 15 ns). However, a slight decrease in the red component (a_t0_* = 14.3; a_t2_* = 13.3) suggests a minor modification of the surface appearance due to probably both some laser-degraded shellac residues and the pigmented substrate. The UV-fluorescence image shows indeed only very local shellac remains. An analogue situation is found following the treatment with the Nd:YAG LQS laser (II). However, only local ablation and film detachment from the surface is achieved with the longer pulse width laser (LQS, 100 ns) as evidenced by the fluorescence image and OCT data. This agrees with a less pronounced photomechanical effect of the laser with the increasing pulse duration. The reduction in the red component is more pronounced (a_t0_* = 14.3; a_t2_* = 10.0) owed mainly to a laser-induced modification of the shellac film that remains on the surface. All the colorimetric coordinates tend to approach the original reference values of the surface before the shellac treatment (t_0_). This is also the case of the last two irradiations with Ho:YAG and Er:YAG lasers (IV and V). The better cleaning results were achieved with the latter, as deduced from the combination of all the data. The ablation is gradual and respectful of the surface characteristics, leaving a thin residual film on the surface, as observed from the UV-induced fluorescence (Figure 5b).

#### 3.2.3. Green Earth

**Figure 6:**
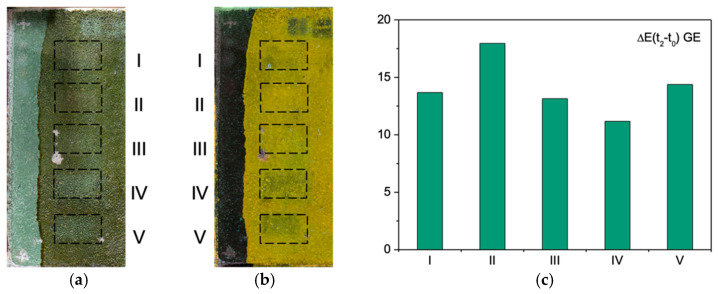
Green Earth specimen as a function of laser treatment (I–V): (**a**) visible image; (**b**) UV-induced visible fluorescence; (**c**) bar plot of ΔE values. Each tested area I–V refers to 1 × 2 cm^2^ area.

Figure 6a,b show the visible and UV-induced fluorescence images of the treated GE sample. The colorimetric parameters of the reference (t_0_), shellac-treated (t_1_), and laser-treated (t_2_) Green Earth mock-up are reported in Table 5. The shellac application induced relevant changes mainly in the L* and b* parameters (ΔL = −13 and Δb = 15.0) with relatively high standard deviation values at t_1_. The latter suggests an inhomogeneous shellac distribution owed probably to the sample morphology.

The laser treatments performed on GE produced quite different effects as compared with the YO and MO samples. First of all, no damage was induced to the substrate, all the colorimetric parameters at t_2_ tend to approach the original paint surface values at t_0_, as evident from Table 5. This can be explained by the high stability of the siliceous Green Earth pigment, as compared to the thermolabile pigments containing iron oxides, such as Yellow Ochre and *Morellone* [30,37,38]. In terms of cleaning efficiency, the Ho:YAG laser (IV) emitting at 2.1 μm showed the best performance, documented by the lowest ΔL*, Δa*, and Δb* values that translate into the lowest ΔE(t_2_−t_0_). This is also confirmed by the UV-induced fluorescence image (Figure 6b) where a reduced fluorescence is observed, especially in sectors IV and V. The cleaning efficiency of all other laser systems (I, III, V) seems comparable in terms of colorimetric coordinates but for the Nd:YAG LQS (II), which shows a very little impact on the surface. 

### 3.3. Thickness Evaluation of the Shellac Removal by OCT

**Figure 7:**
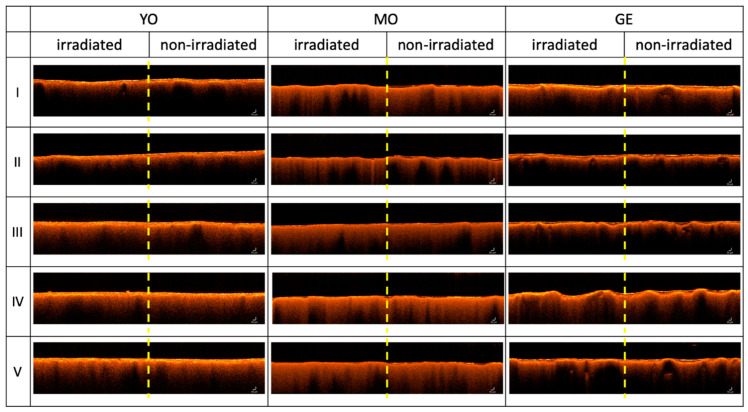
Cross-sectional OCT images (5 mm(x) × 1 mm(z)) as a function of I-V cleaning tests and painted layer (YO, MO, GE). The OCT depth profile images are centered and divided by the yellow dashed line to show the irradiated (**left**) and non-irradiated (**right**) portion of the samples.

The OCT data give complementary information about the impact of laser cleaning, as they can directly reveal the quantity of the material removed and remaining on the surface. The non-invasive OCT method returns stratigraphic images of the sample under investigation. Figure 7 reports such data as a function of the laser cleaning and the paint type recorded on the irradiated and non-irradiated portion for each sample and treatment. As apparent from Figure 7, the varnish settles mainly in the concave portions of the samples. It can also be deduced that the GE sample has the most irregular surface morphology, that translates into the highest maximum varnish thickness value as compared with other samples. Table 6 reports the maximum varnish thickness values (d_max_) before laser treatment to be 16, 21, 27 microns, respectively, for Yellow Ochre, *Morellone* and Green Earth.

Table 6 reports maximum, minimum and mean thickness values of shellac. The standard deviations reflect the spread of the measured values attributable to the inhomogeneous distribution of the polymer on the sample surface. 

OCT data confirm what has been deduced previously by the colorimetric measurements. For the Nd:YAG laser on the MO sample, OCT does not reveal the presence of the superficial film, meaning its thickness is <3.5 μm (below the instrumental detection limit). The same situation is revealed for the Er:YAG laser on the YO specimen. The highest efficiency of the Er:YAG laser is, therefore, confirmed for all the samples but for the GE, where the Ho:YAG laser achieved a higher ablation efficiency (residual shellac thickness 8 μm). The average cleaning rate can be estimated as 1–2 microns/pulse for the Er:YAG laser by considering the initial and the final shellac thickness and 5 passages by the laser as described in the methods. 

## 4. Discussion

Table 7 summarizes the global color change values as a function of all the painted surfaces. The ΔE values in the first row describe the impact of the shellac treatment (t_1_) on the painted surface (t_0_) which is comparable for all the paint types (ΔE~20). The I–V data characterize the effect of laser treatment type (t_2_) as referred to the reference surface (t_0_). Generally speaking, the highest cleaning efficiency has been obtained on the MO sample for all the laser treatments, achieving values of ΔE < 8.4, whereas ΔE values for the YO and GE samples span between 11 and 18.5. The darker substrate of *morellone* facilitates the shellac removal. However, the visible and UV-induced fluorescence images and colorimetric data revealed that the Nd:YAG QS and LQS lasers induced darkening of pigment both in the YO and MO surfaces, being the harmfulness slightly higher in the QS regime for the YO surface. On the other hand, other lasers did not produce significant adverse effects in terms of color changes induced to the substrate. Indeed, the colorimetric parameters of surfaces after III–V cleaning tend to approach their original values at t_0_. The decreasing values of ΔE for the Nd:YAG SFR > Ho:YAG > Er:YAG lasers indicate the highest efficiency for the Er:YAG laser in all the samples but for the GE, where the efficiency seems slightly higher for the Ho:YAG laser. These data are corroborated by the OCT measurements.

The overall results considering all the data from the sensors are summarized in Table 8. It can be seen that the Nd:YAG laser at 1064 nm with Q-switched pulse (15 ns) ablated well in the MO sample and enabled very partial thinning of the film from YO sample, at the cost, however, of some induced damage. The average temperature measured did not account for the instantaneous temperature raise during the very short pulse that could easily reach several hundreds of °C with fast thermal recovery. 

The laser treatment II with the Nd:YAG laser at 1064 nm with long Q-switched pulse (100 ns) is quite similar in its effects as compared with I treatment, with less pronounced adverse effects and a lower photomechanical efficiency.

The laser treatment III with the Nd:YAG laser at 1064 nm with short-free running pulse width (5 μs) enabled only a very partial thinning of the film (OCT residual thickness is 7 and 10 microns, respectively, for the YO and MO samples), as testified also by the colorimetric data. Minimal effects were achieved for the GE sample.

The laser treatment IV with the Ho:YAG laser at 2.1 μm achieved the highest efficiency on the GE sample and a little less efficiency on the other two samples. No damage was induced on any sample. Average temperature is the highest of all the lasers due to both a high fluence and a higher repetition rate (10 Hz).

The laser treatment V with the Er:YAG laser emitting at 2.9 μm with long pulse width (500 μs) proves to be gradual and very versatile as good cleaning results were achieved on all the sample types, enabling steady thinning of shellac layer to about half of its thickness at reasonable ablation rate.

Both the Ho:YAG and the Er:YAG lasers probably act through similar principles involving water vaporization, gas expansion, and micro-distillation phenomena to gradually remove material from the treated surface. The Er:YAG laser acts more efficiently due to a more efficient coupling of the laser radiation with the -OH vibrational modes of the shellac itself.

## 5. Conclusions

Three YAG lasers—doped with Nd, Ho, and Er ions—emitting, respectively, at 1064, 2100, and 2940 nm were tested for the removal of a shellac layer from mural painting mock-ups. The Nd:YAG laser was tested at three different pulse durations: 15 ns, 100 ns, and 5 μs, while for the Ho:YAG and Er:YAG the pulse widths were, respectively, 95 and 500 μs. The lasing conditions were set just above the ablation threshold at frequencies allowing the cleaning of 1 × 2 cm^2^ areas (5 Hz for Nd:YAG and 10 Hz for Ho:YAG and Er:YAG). The laser effects were inspected by four complementary techniques (infrared thermography microscopy, optical coherence tomography, and ultraviolet-visible-infrared imaging). 

The data obtained through the proposed multisensorial approach proved that among all the lasers, the Er:YAG one is the most versatile and gradual in removing the organic layer. For the first time, the Ho:YAG laser efficiency is demonstrated in gradual thinning of a shellac layer while safeguarding the substrate. The two abovementioned treatments require the subsequent cleaning of the mechanically compromised layer with the ethanol-soaked cotton swab. On the other hand, the Nd:YAG QS and LQS lasers at 1064 nm allowed contactless removal only from specific mock-ups, at the cost of local damage. The Nd:YAG SFR at 1064 nm proved very poor efficiency in removal of shellac. The infrared thermography is efficient in monitoring the average surface temperature but is not fast enough to capture the instantaneous temperature rise induced by the short laser pulses. 

## Figures and Tables

**Table 1:** Pigments and their acronyms used in the fresco mock-up.

Pigment Acronym	Pigment Type
YO	Yellow Ochre
MO	Morellone
GE	Green Earth

**Table 2:** Laser parameters used for treatments, all in modality wet.

Treatment No.	Laser Source	Wave-Length λ [nm]	Pulse Width τ	Energy *E* [mJ]	Fluence *F* [J/cm^2^]	Frequency *f* [Hz]
I	Nd:YAG QS	1064	15 ns	60	1.2	5
II	Nd:YAG LQS	1064	100 ns	150	3.1	5
III	Nd:YAG SFR	1064	5 μs	100	2.0	5
IV	Ho:YAG Ablation (short)	2100	95 μs	500	10.2	10
V	Er:YAG Long	2940	500 μs	150	3.1	10

**Table 3:** Colorimetric L*, a*, b* parameters and their standard deviations (std) of Yellow Ochre mock-up: reference t_0_, after shellac application t_1_, after I–V laser treatment t_2_.

Surface	L*	std	a*	std	b*	std	ΔL*	Δa*	Δb*
t_0_	69.0	0.0	10.7	0.6	39.3	2.1			
t_1_	54.0	1.7	18.7	0.6	51.3	1.2	−15.0	8.0	12.0
t_2_ I	51.0	1.0	12.7	0.6	36.3	1.5	−18.0	2.0	−3.0
t_2_ II	53.0	1.0	13.3	0.6	35.7	1.2	−16.0	2.7	−3.7
t_2_ III	58.0	0.0	17.5	0.6	46.0	0.6	−11.0	6.8	6.7
t_2_ IV	58.7	0.6	17.0	0.0	45.7	0.6	−10.3	6.3	6.3
t_2_ V	60.0	1.0	16.0	0.0	43.7	1.2	−9.0	5.3	4.3

**Table 4:** Colorimetric L*, a*, b* parameters and their standard deviations (std) of the *morellone* mock-up: reference t_0_, after shellac application t_1_, after I–V laser treatment t_2_.

Surface	L*	std	a*	std	b*	std	ΔL*	Δa*	Δb*
t_0_	36.3	0.6	14.3	0.6	5.3	0.6			
t_1_	22.3	0.6	21.3	0.6	17.0	1.0	−14.0	7.0	11.7
t_2_ I	33.0	1.0	13.3	0.6	9.0	1.7	−3.3	−1.0	3.7
t_2_ II	33.3	0.6	10.0	0.0	8.7	1.2	−3.0	−4.3	3.3
t_2_ III	29.5	0.6	16.5	0.6	9.5	0.6	−6.8	2.2	4.2
t_2_ IV	30.0	0.0	16.7	0.6	9.3	0.6	−6.3	2.3	4.0
t_2_ V	30.0	1.0	17.0	0.0	8.7	1.2	−6.3	2.7	3.3

**Table 5:** Colorimetric L*, a*, b* parameters and their standard deviations (std) of the Green Earth mock-up: reference t_0_, after shellac application t_1_, after I–V laser treatment t_2_.

Surface	L*	std	a*	std	b*	std	ΔL*	Δa*	Δb*
t_0_	55.7	0.6	−17.0	1.0	13.0	0.0			
t_1_	42.7	4.0	−12.0	0.0	28.0	2.6	−13.0	5.0	15.0
t_2_ I	46.0	1.7	−10.0	1.0	19.7	1.5	−9.7	7.0	6.7
t_2_ II	42.3	0.6	−8.7	1.2	21.7	1.5	−13.3	8.3	8.7
t_2_ III	46.0	1.2	−11.5	0.6	20.0	0.6	−9.7	5.5	7.0
t_2_ IV	46.3	1.2	−12.3	0.6	17.0	0.0	−9.3	4.7	4.0
t_2_ V	42.7	0.6	−12.7	0.6	17.3	0.6	−13.0	4.3	4.3

**Table 6:** Maximum, minimum and mean thickness values (respectively d_max_, d_min_, d_r_) of the shellac layer before and after I–V laser treatments. Standard deviation is a measure of the thickness variability. N.d. stands for not detectable thickness (i.e., below the instrumental limit of detection which is about 3.5 μm in *z*).

Sample	YO			MO			GE		
Thickness [µ]	d_max_–d_min_	d_r_	std	d_max_–d_min_	d_r_	std	d_max_–d_min_	d_r_	std
Non-irradiated	16–7	11	4	21–7	12	5	23–7	15	5
I	16–5	7	3	n.d.	n.d.	n.d.	23–7	14	6
II	9–2	6	2	n.d.	n.d.	n.d.	22–9	14	6
III	12–5	7	3	12–7	10	2	21–7	14	4
IV	12–7	9	2	16–5	11	3	12–5	8	2
V	n.d.	n.d.	n.d.	6–4	6	2	16–5	11	4

**Table 7:** Global color change values (ΔE) as a function of the paint type following the shellac application (t_1_–t_0_) and the I–V laser treatments (t_2_–t_0_).

Treatment	YO	MO	GE
t_1_−t_0_	20.8	19.5	20.5
I	18.4	5.1	13.7
II	16.6	6.2	18.0
III	14.6	8.3	13.1
IV	13.7	7.8	11.2
V	11.3	7.6	13.8

**Table 8:** Overall observations of the effects of the I–V laser treatments (t_2_−t_0_).

**Treatment**	**YO**	**MO**	**GE**
I	Thinning Surface darkening	In-depth ablation Surface darkening	No evident cleaning No substrate modifications
II	Thinning Surface darkening	Delamination/Surface darkening	No evident cleaning No substrate modifications
III	Thinning No substrate modifications	Thinning No substrate modifications	No evident cleaning No substrate modifications
IV	Thinning No substrate modifications	Thinning No substrate modifications	Thinning No substrate modifications
V	Thinning No substrate modifications	Thinning No substrate modifications	Thinning No substrate modifications

## Data Availability

Please write to the authors in case you are interested in accessing/reusing data from this study.
